# Estimating the Burden of Heat‐Related Illness Morbidity Attributable to Anthropogenic Climate Change in North Carolina

**DOI:** 10.1029/2022GH000636

**Published:** 2022-11-01

**Authors:** Jagadeesh Puvvula, Azar M. Abadi, Kathryn C. Conlon, Jared J. Rennie, Stephanie C. Herring, Lauren Thie, Max J. Rudolph, Rebecca Owen, Jesse E. Bell

**Affiliations:** ^1^ Department of Environmental, Agricultural and Occupational Health College of Public Health University of Nebraska Medical Center Omaha NE USA; ^2^ Department of Public Health Sciences University of California Davis Davis CA USA; ^3^ NOAA/National Centers for Environmental Information Asheville NC USA; ^4^ NOAA/National Centers for Environmental Information Boulder CO USA; ^5^ Division of Public Health, Occupational & Environmental Epidemiology North Carolina Department of Health and Human Services Raleigh NC USA; ^6^ Heider College of Business Creighton University Omaha NE USA; ^7^ HealthCare Analytical Solutions, INC Bend OR USA; ^8^ School of Natural Resources University of Nebraska‐Lincoln Lincoln NE USA; ^9^ Daugherty Water for Food Global Institute University of Nebraska Lincoln NE USA

**Keywords:** climate change, climate attribution, climate projections, heat related illness, morbidity

## Abstract

Climate change is known to increase the frequency and intensity of hot days (daily maximum temperature ≥30°C), both globally and locally. Exposure to extreme heat is associated with numerous adverse human health outcomes. This study estimated the burden of heat‐related illness (HRI) attributable to anthropogenic climate change in North Carolina physiographic divisions (Coastal and Piedmont) during the summer months from 2011 to 2016. Additionally, assuming intermediate and high greenhouse gas emission scenarios, future HRI morbidity burden attributable to climate change was estimated. The association between daily maximum temperature and the rate of HRI was evaluated using the Generalized Additive Model. The rate of HRI assuming natural simulations (i.e., absence of greenhouse gas emissions) and future greenhouse gas emission scenarios were predicted to estimate the HRI attributable to climate change. Over 4 years (2011, 2012, 2014, and 2015), we observed a significant decrease in the rate of HRI assuming natural simulations compared to the observed. About 3 out of 20 HRI visits are attributable to anthropogenic climate change in Coastal (13.40% [IQR: −34.90,95.52]) and Piedmont (16.39% [IQR: −35.18,148.26]) regions. During the future periods, the median rate of HRI was significantly higher (78.65%: Coastal and 65.85%: Piedmont), assuming a higher emission scenario than the intermediate emission scenario. We observed significant associations between anthropogenic climate change and adverse human health outcomes. Our findings indicate the need for evidence‐based public health interventions to protect human health from climate‐related exposures, like extreme heat, while minimizing greenhouse gas emissions.

## Key Points

- Quantified the association between daily maximum temperature and heat‐related illness (HRI) emergency room visits
- During 2011–2016, 15% of the HRI emergency room visits in North Carolina were attributable to anthropogenic climate change
- HRI emergencies are projected to increase by 32% during 2036–2065 and 79% during 2070–2099, assuming RCP8.5 emissions compared to RCP4.5

## 1 Introduction

Since the mid‐twentieth century, the frequency and duration of hot days have increased globally due to anthropogenic climate change (Hoegh‐Guldberg et al., 2018). Global temperature is very likely to increase up to 1.5°C–1.6°C during 2021–2040, 1.6°C–2.4°C during 2041–2060, and 1.4°C–4.4°C during 2081–2100, relative to 1850–1900 (IPCC, 2021). These increases in temperature since the mid‐twentieth century are primarily the result of anthropogenic greenhouse gas emissions (IPCC, 2021). Mora, et al. (2017) estimate that roughly 30% of the current global population is exposed to extreme heat conditions; this number is expected to increase to 50%–75% by 2100 (Mora et al., 2017).

Exposure to extreme heat leads to heat‐related morbidity and mortality. Extreme heat outcomes can be characterized as direct (e.g., heat‐related illness [HRI]), or indirect (e.g., exacerbation of cardiovascular, respiratory, renal, endocrine, and mental health conditions) (Bell et al., 2018; Ebi, Capon, et al., 2021; Sarofim et al., 2016). Failure to acclimatize during extreme heat conditions can result in HRI ranging from muscle cramps, heat exhaustion, and heat stroke (Danzl, 2018). HRI, if untreated, can lead to life‐threatening conditions (LoVecchio, 2016; Nemer & Juarez, 2019). In the United States (US), heat‐related fatalities are more common than deaths due to other natural disasters (NOAA, 2019). In the US, there are an average of 702 heat‐related deaths per year (Vaidyanathan et al., 2020). There is a potential imbalance between heat‐related mortality and morbidity, posing an exponentially higher number of heat‐related emergencies. For example, during a 4‐day heat wave event in North Carolina, there were 556 HRI emergencies compared to 1 heat‐related death (NC‐DHHS, 2016). The magnitude of the HRI emergencies compared to mortality demonstrate the need to focus on morbidity compared to mortality.

The role of climate change has been associated with the increasing trend of heat‐related mortality and morbidity (Bell et al., 2018; Christidis et al., 2019; Ebi, Vanos, et al., 2021). Evidence‐based climate detection and attribution play a key role in characterizing the changes in natural climate variability that are attributable to human activities (Ebi et al., 2017, 2020). There is strong evidence supporting the association between future climate change and mortality (Conlon et al., 2016; Gosling et al., 2017; Guo et al., 2018; Lay et al., 2018). At the same time, attribution of human health risk to anthropogenic climate change is limited to considering mortality as a health outcome (Mitchell et al., 2016; Vicedo‐Cabrera et al., 2021).

It is common for attribution analyses to investigate heat‐related mortality, providing insight into the magnitude of extreme heat events on the most serious health outcomes. Based on the contrast between the frequency of heat‐related mortality and morbidity in North Carolina, we hypothesized that using heat‐related mortality would be an underestimate to quantify human health risks associated with climate change. We investigate heat‐related morbidity to better understand the scope of human health burden associated with climate change. This study estimated the HRI attributable to anthropogenic climate change. Additionally, future HRI burden attributable to climate change was estimated using the climate projections driven by representative concentration pathways (RCPs). The RCPs are greenhouse gas concentration trajectories adopted by the IPCC that are used for climate modeling (IPCC, 2014). Future climate change is typically represented using four RCP scenarios, RCP2.6 is a stringent mitigation scenario, RCP4.5 and RCP6.0 are intermediate scenarios, and RCP8.5 is a scenario with very high greenhouse gas emissions (IPCC, 2014). This study includes two of the four RCP scenarios, comparing the intermediate emission scenario (RCP4.5) and higher emission scenario (RCP8.5).

This study is structured into three sections. In the first section, we aim to evaluate the association between heat metrics and HRI morbidity to estimate the HRI morbidity attributable to climate change. In the second section, we estimated the HRI attributable to the current (2011, 2012, 2014, and 2015) levels of anthropogenic climate change. The third section includes estimating the HRI associated with future climate change under two greenhouse gas emission scenarios (RCPs).

## 2 Materials and Methods

This study includes three analytic components: (a) Modeling and optimization of an epidemiologic model to estimate the rate of HRI emergency department visits, (b) Estimating the HRI burden attributable to current anthropogenic climate change, and (c) Quantifying the HRI burden associated with future climate change.

### 2.1 Study Area

North Carolina has three physiographic regions with distinct climatological profiles: Coastal, Piedmont, and Mountain regions. The Coastal region includes 41 counties, Piedmont consists of 34 counties, and the Mountain contains 25 counties. Due to distinct weather conditions experienced by the population living in these physiographic regions, most of the heat‐related research has been conducted using these sub‐divisions (NC‐DHHS, 2015). The study period includes summer months (1 May to 30 September) over 5 years from 2011 to 2016. Data for 2013 were unavailable and is excluded from the analysis.

### 2.2 HRI Morbidity Data

The HRI data were obtained from the North Carolina Department of Health and Human Services (NC DHHS) that has partnered with 124 hospitals to collect statewide emergency department (ED) visit data to provide real‐time, electronic public health surveillance, which is stored in the North Carolina Disease Tracking and Epidemiologic Collection Tool (NC DETECT, 2021). Heat‐related illnesses were identified using ICD‐9 CM codes with E992/E900.0/E900.0/E900; ICD‐10 CM codes within T67/X30/X32; and various keywords from the chief complaint and triage notes (Harduar Morano & Waller, 2017; NC‐DETECT, n.d). We obtained daily aggregated counts of HRI ED visits. Days with fewer than five HRI cases were suppressed to maintain the confidentiality of patient identifiable information.

Decennial census population data at the county level from 2010 were aggregated to the three regions in North Carolina. Equation 1 was used to estimate the rate of HRI morbidity per study region.

(1) $$HRI\;morbidity\;rate=\frac{Count\;HRI\;emergency\;department\;visits}{population\;at\;risk}*100,000$$

### 2.3 Observed Meteorological Data

Daily temperature data (mean (*t*_mean_), minimum (*t*_min_), and maximum (*t*_max_)) from the Global Historical Climatology Network‐Daily (GHCN‐D) database were extracted and aggregated by region. The GHCN‐D data set contains daily temperature measurements based on approximately five stations per region (Houston et al., 2012; NOAA, n.d). Daily temperature measurements were homogenized to account for instrumentation and processing station observations (Rennie et al., 2019). Dew point data was obtained from the Parameter‐elevation Regressions on Independent Slopes Model (PRISM) data set (Model, 2019). The relative humidity (RH), maximum apparent temperature (MAT), US NWS/Steadman's heat index (NWS_HI & Steadmans_HI), humidex, thermal discomfort index (TDI), and Excess Heat Factor (EHF) were computed by study region (Anderson et al., 2013; Baccini et al., 2008; Castelhano; & Laboclima, 2017; Langlois et al., 2013; PRISM, n.d).

### 2.4 Natural Simulations

The natural simulations are an estimate of daily maximum temperature (*t*_max‐NS_) in the absence of human‐caused greenhouse gas emissions. The natural simulations are based on the greenhouse gas emissions similar to the preindustrial period (1980s) and not adjusted for stratospheric aerosol burden, solar luminosity (Stone et al., 2019). The daily maximum temperature observations assuming the absence of human‐caused greenhouse gas emissions (similar to preindustrial period) were obtained from the data set developed by the Climate of the twentieth Century Plus Detection and Attribution (C20C + D&A) Project (C20C + D&A, n.d, Stone et al., 2019). The C20C + D&A project is built on an ensemble of multiple dynamic models based on the atmosphere‐land system. Due to lack of data, we excluded the year 2013 and 2016 in the analysis. We extracted daily maximum temperatures during the summer seasons of 2011, 2012, 2014, and 2015.

### 2.5 Climate Projections

Localized and bias‐corrected climate projections were obtained from the Localized Constructed Analogs (LOCA) database (LOCA, n.d; Pierce et al., 2014). The LOCA data set is statistically downscaled from the Climate Model Intercomparison Project 5 (CMIP5) and corrected for bias using constructed analogs (Pierce et al., 2014). The current study is based on study regions, amounting to more coarse geographies. The use of LOCA data with 1/16° resolution allowed us to assign localized temperature projections to finer geographies. We focused on the Community Climate System Model version‐4.0 (CCSM4) and Geophysical Fluid Dynamics Laboratory (GFDL) model outputs as these models were outperformed compared to other climate models in the Southeastern United States (Zhang et al., 2013).

The climate projection data set contains maximum temperature aggregated for three time periods: (a) Baseline (2011–2016), (b) Mid‐century (2036–2065), and (c) Late century (2070–2099). The maximum temperature (t_max‐FS_) was estimated for each period assuming intermediate (RCP4.5) and high‐emission (RCP8.5) scenarios.

### 2.6 Analysis

The analytic data set contains the rate of HRI, *t*_mean_, *t*_min_, *t*_max_, RH, MAT, NWS_HI, Steadman_HI, humidex, TDI, and EHF at a daily scale (S 1). Additionally, we created a nominal variable to describe 7 days of the week (DOW), a binary variable to identify weekend or weekday (Wday), as well as a nominal variable representing month and year.

#### 2.6.1 Evaluating the Relationship Between Heat Metrics and HRI Morbidity

Spearman correlations were run to determine temperature metrics to include in further analysis. Five of 10 variables (*t*_max_, *t*_mean_, NWS_HI, Steadmans_HI, and MAT) had a correlation coefficient greater than 55% and these metrics were considered for evaluating association with HRI (S 2). The exploratory analysis suggested a nonlinear relationship between the rate of HRI and heat metrics. The nonlinear relationship between the HRI rate and heat metrics was evaluated using the Generalized Additive Model (GAM)—“mgcv” package version 1.8–34 (Wood, 2021) and distributed lag nonlinear model (DLNM) approach (the “dlnm” R package version 2.4.5, Gasparrini et al., 2021).

GAM is a semi‐parametric framework to address the nonlinearity using smoothing splines (Dominici & Peng, 2008). Smoothing term was considered for the heat metric using cubic regression bases, with up to 5 knots (Wood, 2017b). The number of smoothing parameters for heat metrics was estimation using Generalized Cross Validation (GCV) method (Wood, 2017b). The associations were evaluated assuming gamma distribution and log link (Wood, 2017a). Additionally, we included day of week, month, and year, as covariates to adjust for potential temporal autocorrelation. To identify a heat metric as a predictor in our nested model with a smaller residual sum of square, we compared the Akaike Information Criterion (AIC) and *R*‐squared values. The statistical model using *t*_max_ outperformed to estimate the rate of HRI compared to other heat metrics (Equation 2).

(2) $$\log\left(E\left[HRI\;morbidity\right]\right)=\sum_{j=1}^{n}b_{j}\left(t_{\max}\right)\beta_{j}+wDay+Month+Year+\varepsilon$$

The distributive effect of heat metrics on HRI was estimated using DLNM (Gasparrini, 2011). The DLNM follows an interrupted time series approach, where daily HRI ED visits were assumed to follow the Poisson distribution and were fit using the GAM, controlling for seasonal effect. We smoothed the heat metric exposure variable using cubic splines with 5 knots at equally spaced heat metric values. Using the “crossbasis” function, we create a basis matrix between the heat metric and 5 lag days to model the association in each dimension. The association between heat metrics and HRI was evaluated for up to 5 lag days. DLNM was implemented using the “gam” function, with daily HRI count as the outcome and the heat metric cross‐basis matrix. The DLNM approach was implemented using “dlnm” package version 2.4.5, and dependencies using “spline,” “mgcv” packages (Gasparrini et al., 2021).

We then estimated the relative risk to daily maximum temperature by 0–5 lag days using the “crosspred” function. The risk estimates for daily maximum temperature were predicted using the 70th percentile value (32°C) as a reference.

#### 2.6.2 Attributing the Burden of HRI Morbidity to Current Anthropogenic Climate Change

The statistical model was trained by physiographic regions (Equation 2.) using *t*_max_, time series variables to estimate the rate of HRI. The model performance metrics were optimum while using three cubic regression splines for *t*_max_ for the Coastal region and four for Piedmont. The daily rate of HRI was estimated corresponding to the daily t_max‐NS_ values. The percentage difference between the observed and estimated HRI rates assuming natural simulation was considered as the burden of HRI attributable to climate change. The mean difference between the daily rate of HRI between observed and natural simulation was tested using paired *t*‐test. Additionally, the frequency of hot days between natural simulations and actual observations was compared using the chi‐square test. The percent of HRI attributable to anthropogenic climate change is expressed as median percent per year and interquartile range (IQR).

#### 2.6.3 Projecting HRI Under Future Climate Change Scenarios

Using *t*_max_ as a predictor, we trained a statistical model (Equation 3.) by physiographic regions to estimate the rate of HRI. Future HRI was estimated over three different 30‐year periods (baseline, mid‐century, and late century), focusing on RCP4.5 and RCP8.5 scenarios. The difference between HRI across two emission scenarios was evaluated using paired *t*‐test and the differences between HRI across the three time periods were assessed using Analysis of variance.

(3) $$\mathrm{Log}\left(E\left[HRI\;morbidity\right]\right)=\sum_{j=1}^{n}b_{j}\left(t_{\max}\right)\beta_{j}+\varepsilon$$

## 3 Results

During the study period, 28.81% (219) of Coastal and 28.94% (220) of Piedmont regional observations were suppressed. The suppressed data were imputed with the median value (3) of the suppressed range. The Mountain region was excluded from the study due to poor data quality (50.13% [381] suppressed).

The mean HRI rate was 54.52 per 100,000 and 34.27 in the Coastal and Piedmont regions, respectively. The annual HRI rate was consistently higher in the Coastal region than in Piedmont. In both study regions, the rate of HRI was higher (Coastal: 40.94% higher; Piedmont: 28.47% higher) during the summer of 2015, compared to the study period (Table 1). The increase in the rate of HRI in 2015 could be due to a 14‐day heat event with *t*_max_ exceeding 32°C, from 13 June to 27 June 2015.

**Table 1:** Rate of HRI Morbidity per 100,000 Persons

	2011	2012	2014	2015	2016	Study period
Coastal	57.71	50.05	35.46	82.59	72.96	54.52
Piedmont	39.12	33.66	19.00	45.69	45.22	34.27

*Note*. The population at risk was estimated using 2010 decennial population estimates per physiographic region in North Carolina.

### 3.1 Association Between Daily Maximum Temperature and HRI

The nonlinear association between HRI and *t*_max_ was established using the GAM (S 3‐A). From the model diagnostics, we observed that about 80% of the deviance in HRI could be explained by Equation 2 (S 3‐B). Due to a higher number of observations with *t*_max_ between 26.7°C and 35°C, there is a narrow residual confidence interval that reflects lower prediction uncertainty.

The results from the DLNM suggest that the relative risk of HRI significantly increased with *t*_max_ of more than 35°C. The HRI relative risk was higher during the day of exposure than the following days (S 4). When the *t*_max_ was recorded as 35°C, the HRI relative risk declined from about 2 to 1.2 from the day of exposure to the following day, potentially indicating a harvesting—or displacement—effect. It showed non‐significant results during lag 2–5 days in the Coastal and Piedmont regions (S 5). The results from the DLNM suggest a negligible distributive effect of daily maximum temperature exposure on HRI morbidity.

As the HRI risk was higher during the day of exposure than in the latent period, further analysis is based on the same‐day exposure‐outcome relationship. As the primary goal of the current work was to build a prediction model rather effect estimation, the GAM (Equations 2 and 3) was used to estimate the rate of HRI.

### 3.2 Burden of HRI Attributable to Anthropogenic Climate Change

**Figure 1:**
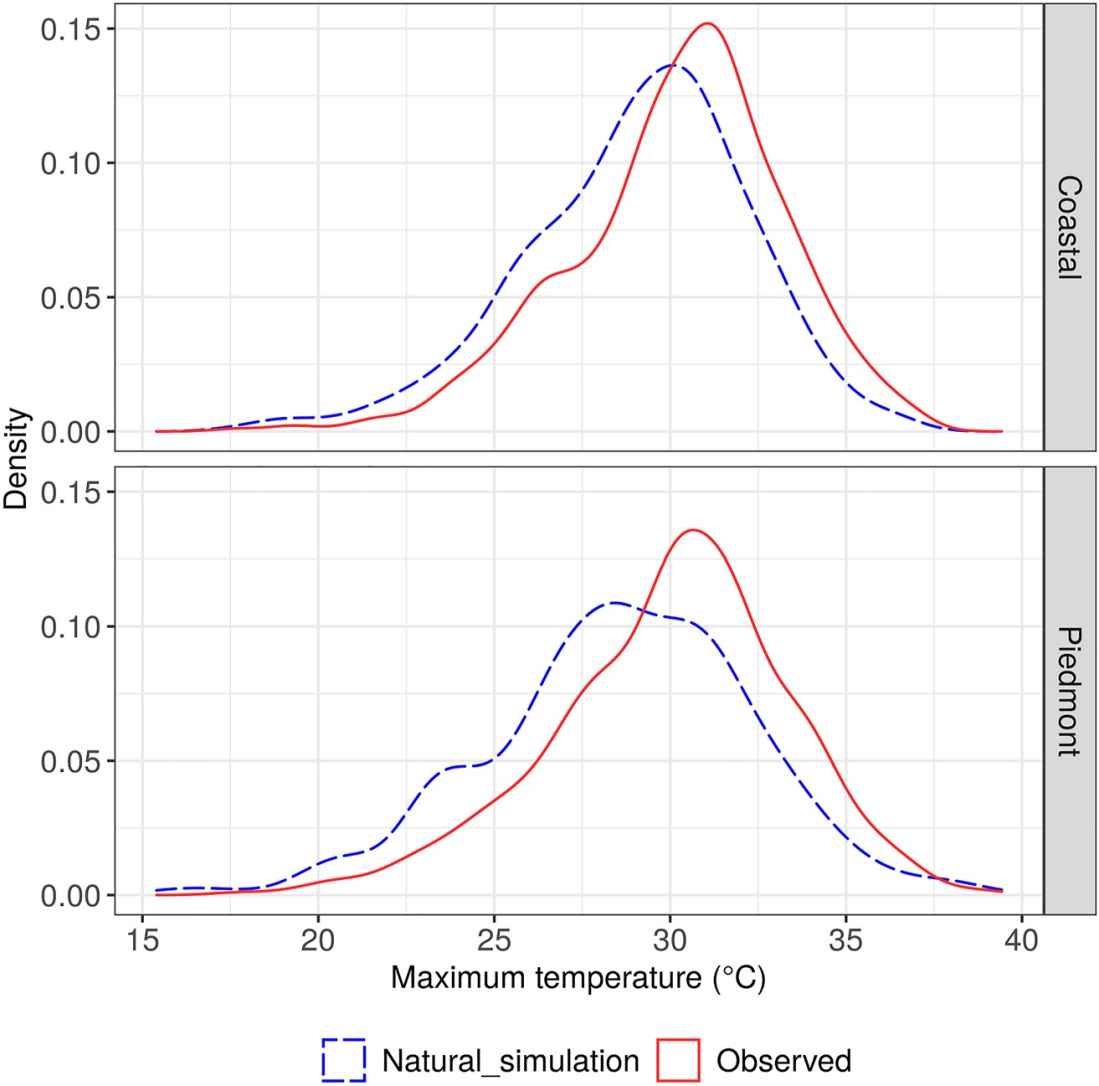
Comparison of the daily maximum temperature (actual observations vs. natural scenario). Observed data were obtained from the GHCN‐D database. Simulated daily temperature data assuming without anthropogenic climate change (natural simulation) were obtained from the Climate of the twentieth Century Plus Detection and Attribution (C20C+D&A) Project.

**Figure 2:**
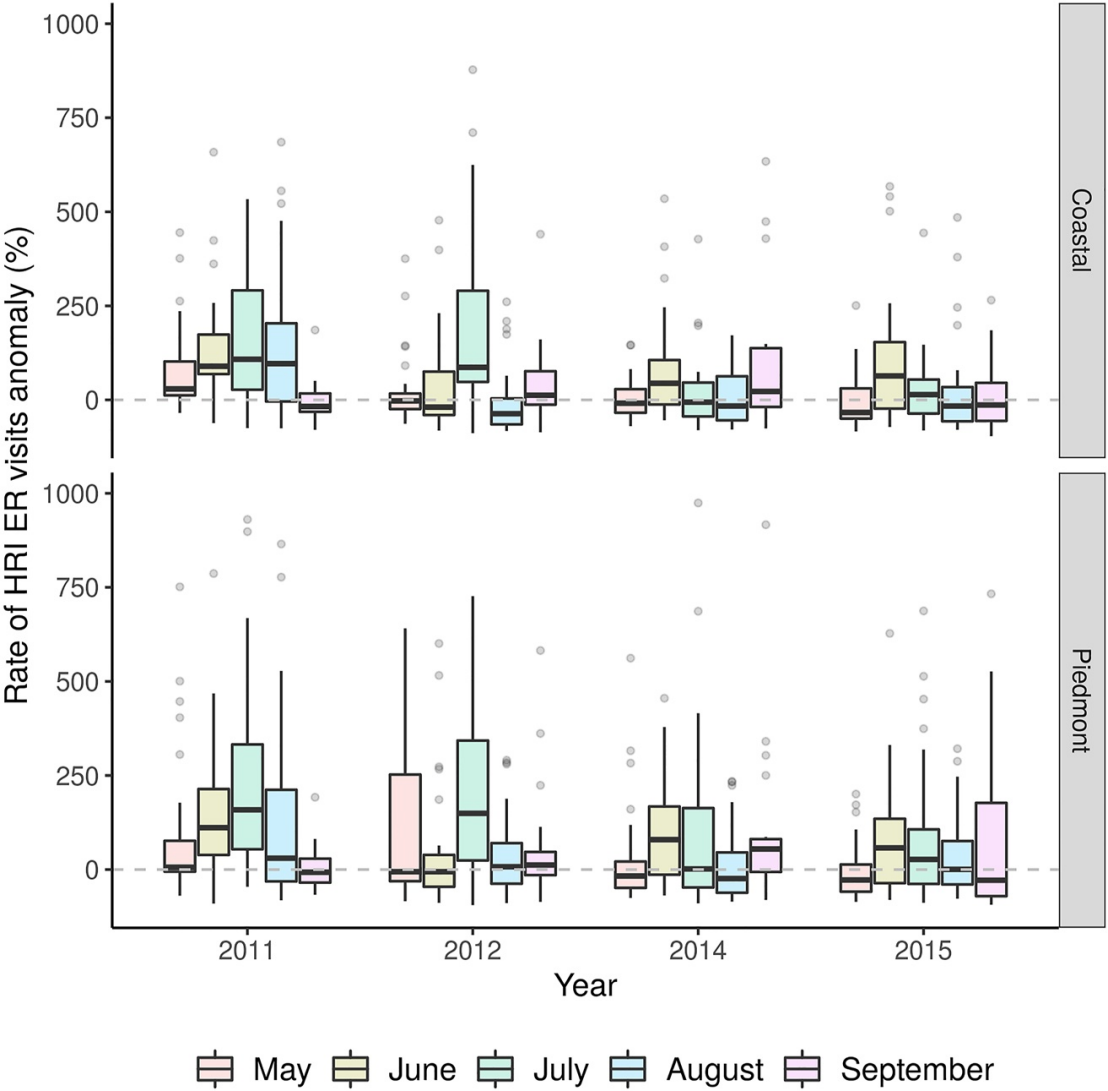
Heat‐related illness (HRI) morbidity attributable to anthropogenic climate change. Box plots are color coded by summer months and grouped by year. The boxplots show the median and interquartile range (25%–75% range), outliers were represented using gray dots. The proportion of the box plot above the horizontal dashed line represent percent increase in HRI morbidity attributable to climate change.

Over the 4 years studied (2011, 2012, 2014, and 2015), the frequency of hot days was about 30% higher in both the Coastal and Piedmont regions, among the actual observations than natural simulations (*p‐*value < 0.001) (Figure 1). We observed a significant reduction in the daily mean rate of HRI morbidity in the Coastal (estimated assuming natural simulation: 0.32 per 100,000, observed: 0.40 per 100,000; *p‐*value < 0.001) and Piedmont (estimated assuming natural simulation: 0.19 per 100,000, observed: 0.24 per 100,000; *p‐*value < 0.001) assuming natural scenario than actual observations. In the Coastal region, 13.40% (IQR: −34.90,95.52) of the HRI morbidity is attributable to anthropogenic climate change and 16.39% (IQR: −35.18,148.26) in the Piedmont region (Figure 2). Based on our attribution analysis, about 83 HRI ED visits per summer season (152 days) in the Coastal region and 85 in Piedmont could be attributed to anthropogenic climate change.

### 3.3 Burden of HRI Morbidity in Context of Future Climate Change

Aggregate *t*_max_ values for the baseline, mid‐century, and late‐century were estimated assuming intermediate emission (RCP4.5) and higher emission (RCP8.5) scenarios using the CCSM4 and GFDL‐ESM2M model outputs. In both the Coastal and Piedmont regions, we observed a significant increase in the median HRI.

**Figure 3:**
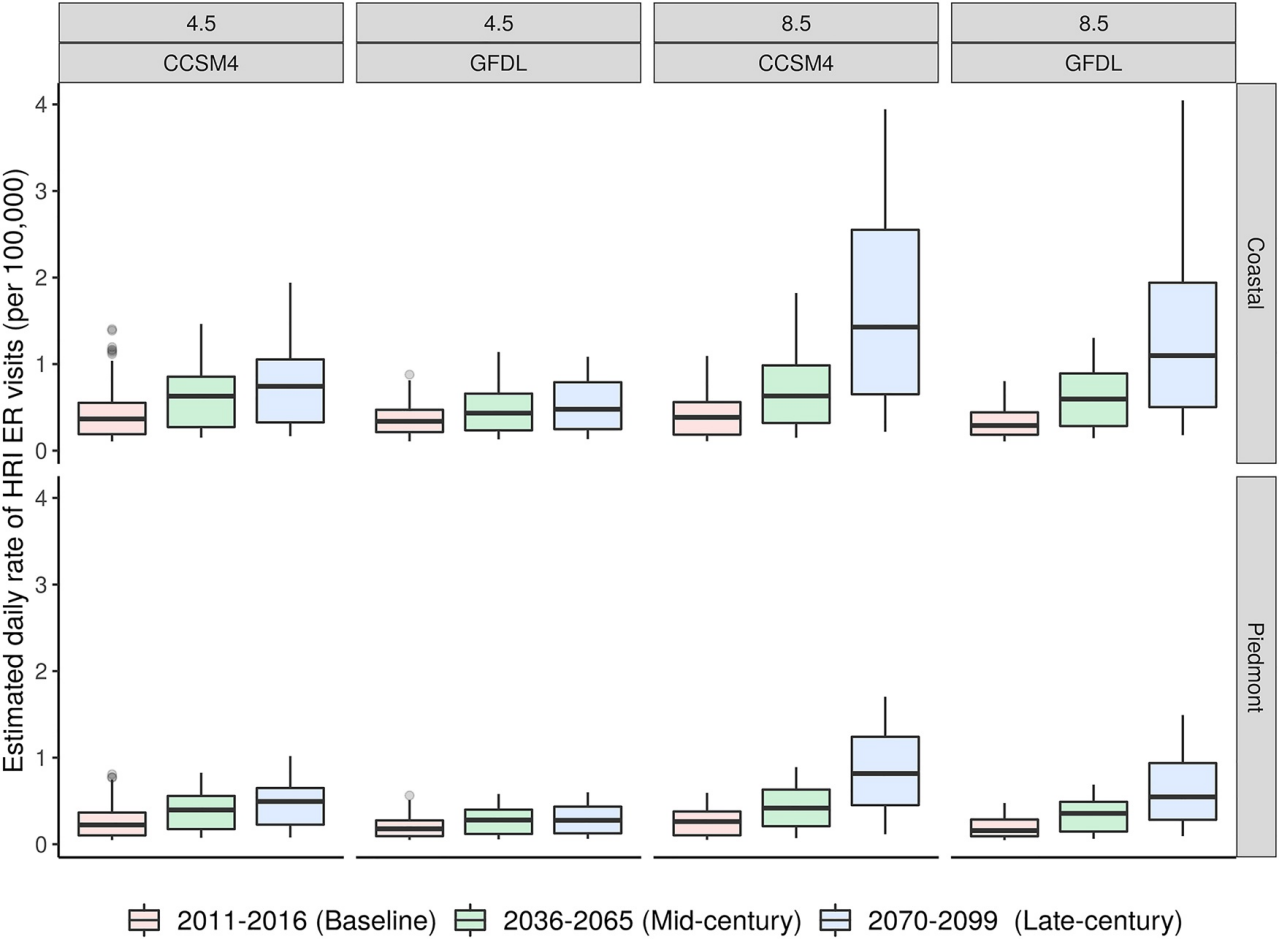
Estimated heat‐related illness (HRI) morbidity rate in context of climate change. The box plots are color coded by time‐period and grouped by climate scenario/climate model. The box plot shows median and interquartile range (25%–75%), with outliers using gray dots. CCSM4—Community Climate System Model (version. 4) and GFDL—Geophysical Fluid Dynamics Laboratory.

In the Coastal region, during the mid‐century, we observed up to 31.45% increase in the median HRI assuming a higher emission scenario compared to intermediate (*p‐*value < 0.001). In the late century, the median HRI increased up to 78.65%, assuming higher emission scenario, compared to the intermediate scenario (*p‐*value < 0.001). Additionally, assuming the intermediate emission scenario, the median HRI increased up to 53.01% during the mid‐century and up to 67.98% in late century, compared to the baseline period (*p‐*value < 0.001). Similarly, assuming a higher emission scenario, the median HRI increased up to 68.77% during the mid‐century and up to 116.31% in the late century, compared to the baseline (*p‐*value < 0.001) (Figure 3).

In the Piedmont region, during the mid‐century, the median HRI increased up to 24.17% assuming higher emission, compared to intermediate (*p‐*value < 0.001). In the late century, the median HRI increased up to 65.85% assuming higher emissions, compared to intermediate emission scenario (*p‐*value < 0.001). Additionally, assuming intermediate emissions, the median HRI increased up to 55.89% during the mid‐century and up to 75.59% in late century, compared to the baseline (*p‐*value < 0.001). Assuming higher emission scenario, the median HRI increased up to 77.28% during mid‐century and up to 110.35% in late century, compared to the base line (*p‐*value < 0.001).

## 4 Discussion

To our knowledge, this is the first study to identify a significant increase in HRI associated with current and future anthropogenic climate change. We identified that anthropogenic climate change attributed to higher frequency of hot days in North Carolina over a 4‐year period from 2011 to 2016. During the 4‐year study period, our findings suggest about 3 out of 20 HRI emergency department visits in North Carolina were attributable to anthropogenic climate change. In addition, future projections of climate change showed a continued increase in HRI over the next century.

Our findings are similar to other studies that have found an increase in heat‐related mortality attributable to anthropogenic climate change. However, this is the first study to focus on morbidity. Unlike studies focused on mortality, morbidity better captures the total human health burden associated with climate change. Other studies focused on health outcomes associated with climate‐related events show that mortality often underestimates the total impacts on health and the associated healthcare costs. Based on our results in two regions in North Carolina, we estimate that 13.4% and 16.4% of HRI emergency room visits from 2011 to 2016 occurred because of anthropogenic climate change. The HRI attributable to anthropogenic climate change could be translated to an average of up to 85 HRI emergency department visits per physiographic region per summer season. Similarly, Vicedo‐Cabrera et al. (2021) reported an average of about 23 heat‐related deaths per year in 6 major cities in North Carolina attributable to anthropogenic climate change (Vicedo‐Cabrera et al., 2021). Our results suggest that heat‐related morbidity is 3.69 times higher than the heat‐related mortality rate reported by Vicedo‐Cabrera et al. (2021). The higher number of HRI could be due to the difference in the total number of cities included in our study (all the area covered under Coastal and Piedmont regions) compared to Vicedo‐Cabrera et al., 2021, which included 6 major cities in North Carolina. It is also natural that morbidity should be higher than estimates of mortality, as not every heat‐related illness results in a death. In North Carolina, the annual mean heat‐related mortality rate is about 200 times less than the rate of heat‐related ED visits (0.11 [1997–2001] vs. 22.2 [2007–2012]) per 100,000 persons) (Mirabelli & Richardson, 2005; Sugg et al., 2016). Additionally, the natural simulation data used in this study is from the C20C + D&A project (Stone et al., 2019), whereas their simulation runs were obtained from the Detection and Attribution Model Intercomparison Project (DAMIP). The natural simulations from C20C + D&A were based dynamical ocean model (ocean surface and sea ice conditions), and are hypothesized to have minimal bias than the simulations from DAMIP that are based on prescribed sea surface conditions (Stone et al., 2019). The use of exposure data in our study from the C20C + D&A project versus DAMIP data being used by Vicedo‐Cabrera et al., 2021, could introduce heterogeneity in ambient heat exposure assessment. We observed a significant decline in the frequency of hot days assuming natural simulations than the actual observations, similar to Mitchell et al. (2016) and Vicedo‐Cabrera et al. (2021). The HRI rate was significantly higher during the actual observations than in natural simulations. Further studies need to explore the current attributable impacts on morbidity associated anthropogenic climate change.

Along with the current climate risk attribution, we estimated the HRI associated with the future climate change. This study discussed the burden of HRI associated with climate change by comparing the HRI rate assuming intermediate and high emission scenarios. We observed a significant increase (up to 78.65% in Coastal and 65.85% in Piedmont regions) in HRI assuming a higher emission scenario (RCP8.5) compared to the intermediate emission scenario (RCP4.5). Additionally, the median rate of HRI significantly increased during the mid (up to 68.77% in Coastal and 77.28% in Piedmont) and late century (up to 116.31% in Coastal and 110.35% in Piedmont) compared to the baseline during both the emission scenarios. Similar results were reported by Lay et al. (2018), who estimated an increase in HRI emergencies by 32% in 2050 and 79% in 2090, assuming RCP8.5 compared to the RCP4.5 scenario. Kingsley et al. (2016) reported a 20% increase in HRI in Rhode Island assuming the RCP8.5 scenario and attributable to climate change. Our results (up to 31.45% increase in HRI during mid‐century and 78.65% in the late century) are similar to the HRI changes reported by Lay et al. (2018) and Kingsley et al. (2016). The heterogeneity in the findings from our study compared to the literature could be explained by the climate variability, human vulnerability to natural hazards across geographies (Ebi et al., 2018). The findings from the fourth national climate assessment suggest that the population living the Southeastern United States are exposed to extreme temperatures than the other parts of the US (Carter et al., 2018). Similarly, variability in vulnerability characteristics across geographies that interact with natural hazards such as extreme heat, could result in differential exposure‐response associations by geographic areas (Berke et al., 2015; Cutter & Finch, 2008). Transitioning to the presentation of the HRI burden associated with anthropogenic climate change, we discussed our findings using the percent increase in HRI morbidity. Lay et al. (2018) estimated the attributable cost of heat on morbidity by exploring employer‐based health insurance claims database of people under the age of 65. Discussing HRI in terms of cost often provides compelling insights that would effectively advocate policy change but were associated with limitations. The health data being used by Lay et al. (2018) excluded the most vulnerable population groups, such as the unemployed and elderly. In comparison, our study did not restrict vulnerable population groups from North Carolina.

The evidence‐based findings from our study discussing HRI attributable to climate change play a key role in public health education and preparedness that are relevant to extreme temperature exposure. Translating our results into public health action by developing community scale risk mitigation plans could substantially minimize the HRI risk. In addition, this study could support actuaries as a framework to assess the human health risks associated with extreme events driven by climate change. Unlike the existing literature, our methodology contextualized both the current and future HRI morbidity attributable to climate change. Our comprehensive methodologic approach in quantifying HRI morbidity associated with climate change using the acute (GAM) and distributive (DLNM) associations, would allow direct comparison of effect estimates from two statistical approaches that are commonly practiced in climate attribution studies. Additionally, population vulnerabilities such as age, gender, comorbidities, household type, income, nature of the employment, and daily activity, are known to interact or mediate with temperature exposure in exacerbating HRI risk (Ebi, Vanos, et al., 2021). Along with population vulnerabilities, community build characteristics could mediate the HRI risk associated with climate change. Certain phenomenon such as the urban heat island and heat dome effect, were identified to be driving factors associated with extreme heat exposure disparities by geography (Henderson et al., 2022; Tuholske et al., 2021). These phenomena are typically driven by neighborhood characteristics such as land use and land cover (Fard et al., 2021). Further studies discussing human health risks attributable to the current and future climate change, by considering population vulnerabilities and neighborhood characteristics could address these gaps in the research. As we observed strong correlation with daily maximum temperature, compared to the heat metrics based on temperature and humidity during the study period, the statistical model based on temperature was trained to predict HRI morbidity attributable to current and future climate change. According to the 2017 National Climate Assessment, there is a minimal change in total annual summer precipitation associated with climate change in the Southeastern region, compared to the other regions in the US (Easterling et al., 2017). However, the future changes in precipitation trends could influence the HRI morbidity estimates. In this paper, we did not discuss relative humidity due to the large uncertainties that make future estimates less reliable (Flato et al., 2013; Risi et al., 2012).

In this study, the future projections of HRI were estimated using static population (2010 decennial census). The objective of this study is to estimate the percent change in HRI over time, rather than presenting an absolute count of future HRI ED visits. Few studies adjusted for future population growth (Lay et al., 2018; Martinez et al., 2016) to describe the results based on absolute counts to estimate the cost associated with hospitalizations and ED visits. Estimation of the future population growth would be essential to translate estimated number of HRI morbidity or mortality from the current period to project future HRI. In this study, we discussed the human health burden associated with future climate change using the percent change in the rate of HRI, which do not require population growth estimation. Due to data limitations, we did not calculate the excess number of morbidities associated with future climate change, which is essential for the cost estimation. Additionally, changes in population characteristics across North Carolina physiographic divisions could influence our study results.

## 5 Conclusions

This work adds strong evidence quantifying the human health risk associated with current and future climate change in the Southeastern United States. This study estimated about 3 out of 20 emergency room visits associated with HRI in North Carolina during the study period is attributable to anthropogenic climate change. Additionally, a substantial increase in HRI assumed a high emission scenario compared to an intermediate emission scenario. Our findings suggest that anthropogenic climate change is already having a significant effect on human health and will continue to have impacts in the future. Our findings suggest that adaptation interventions, along with greenhouse gas mitigation, are needed to reduce the health impacts of climate change. As current climate change is already causing increases in hospitalization, public health interventions should be implemented now to reduce the current and future health burden. By using best knowledge and practices, the health impacts associated with climate change can be addressed.

## Conflict of Interest

The authors declare no conflicts of interest relevant to this study.

## Supporting information

Supporting Information S1

## Data Availability

Observed temperature data for research are available at the Global Historical Climatology Network daily (GHCNd) database, [Data Set]. Available at: https://www.ncei.noaa.gov/products/land-based-station/global-historical-climatology-network-daily
Observed precipitation and dew point data for research are available at the PRISM Climate Group database, [Data set]. Available at: https://www.prism.oregonstate.edu/recent/
The natural simulations data for research are available at the C20C + Detection and Attribution Project database, [Data set]. Available at: https://portal.nersc.gov/cascade/data/downloader.php?get_dirs=
The future climate simulation data for research are available at the Localized Statistical Downscaling Constructed Analogs database, [Data Set]. Available at: http://loca.ucsd.edu/
The heat‐related emergency department visit data are not publicly available, but available to researchers through the North Carolina Department of Health and Human Services. Available at: https://ncdetect.org/data/.
