# Supplementary material for: Estimating the Burden of Heat‐Related Illness Morbidity Attributable to Anthropogenic Climate Change in North Carolina
Source: Geohealth. 2022 Nov 1;6(11):e2022GH000636. doi: 10.1029/2022GH000636 (PMC9685474; doi:10.1029/2022GH000636)
Supplement: Supplementary file 1 — Supporting Information S1 [file GH2-6-e2022GH000636-s001.docx]

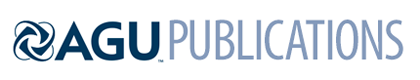


*GeoHealth*

Supporting Information for

**Estimating the Burden of Heat-related illness morbidity Attributable to Anthropogenic Climate Change in North Carolina**

Jagadeesh Puvvula^1^, Azar M. Abadi^1^, Kathryn C. Conlon^2^, Jared J. Rennie^3^, Stephanie C. Herring^4^, Lauren Thie^5^, Max J. Rudolph^6^, Rebecca Owen^7^, Jesse E. Bell^1,8,9^

^1^Department of Environmental, Agricultural and Occupational Health, College of Public Health, University of Nebraska Medical Center, Omaha, NE, 68198 USA

^2^Department of Public Health Sciences, University of California Davis, One Shields Ave, Davis CA, 95616 USA

^3^ NOAA/National Centers for Environmental Information, Asheville, NC, 28801 USA

^4^NOAA/National Centers for Environmental Information, Boulder, CO USA

^5^Division of Public Health, Occupational & Environmental Epidemiology, North Carolina Department of Health and Human Services, Raleigh, NC, 27699 USA

^6^Heider College of Business, Creighton University, Omaha, NE, 68102 USA

^7^HealthCare Analytical Solutions, INC., Bend, OR, 97701 USA

^8^School of Natural Resources, University of Nebraska-Lincoln, Lincoln, NE, 68583 USA

^9^Daugherty Water for Food Global Institute, University of Nebraska, Lincoln, NE, 68588 USA

**Contents of this file**

Figures S2 - S6

Tables S1 and S3-B

| **Variables** | **Source** |
| --- | --- |
|  |  |
| Temperature (t_max_, t_min_, t_mean_) | GHCN-D |
| Dew point | PRISM |
| Relative humidity | Calculated using daily maximum temperature and dew point. |
| Humidex/Heat Index | Calculated using daily maximum temperature and relative humidity |
| Thermal Discomfort index | Calculated using daily maximum temperature and relative humidity |
| Excess Heat Factor | Calculated using the daily maximum temperature |
| Natural simulations | C20C+D&A |
| Future climate projections | LOCA |
| HRI emergency department visits | NC DETECT |
| Total population | US Census Bureau |

Table S1.

Variables and data sources. All the variables below (except total population) were measured at a daily scale by the physiographic region

Figure S2. Association between meteorological variables/heat indices and HRI morbidity (A-Coastal & B-Piedmont). The numeric values corresponding to the color legend (blue-positive association and red-negative association) are the Spearman correlation coefficients that describe the association between two variables. The non-significant (P<0.05) correlation coefficient values were stricken off.


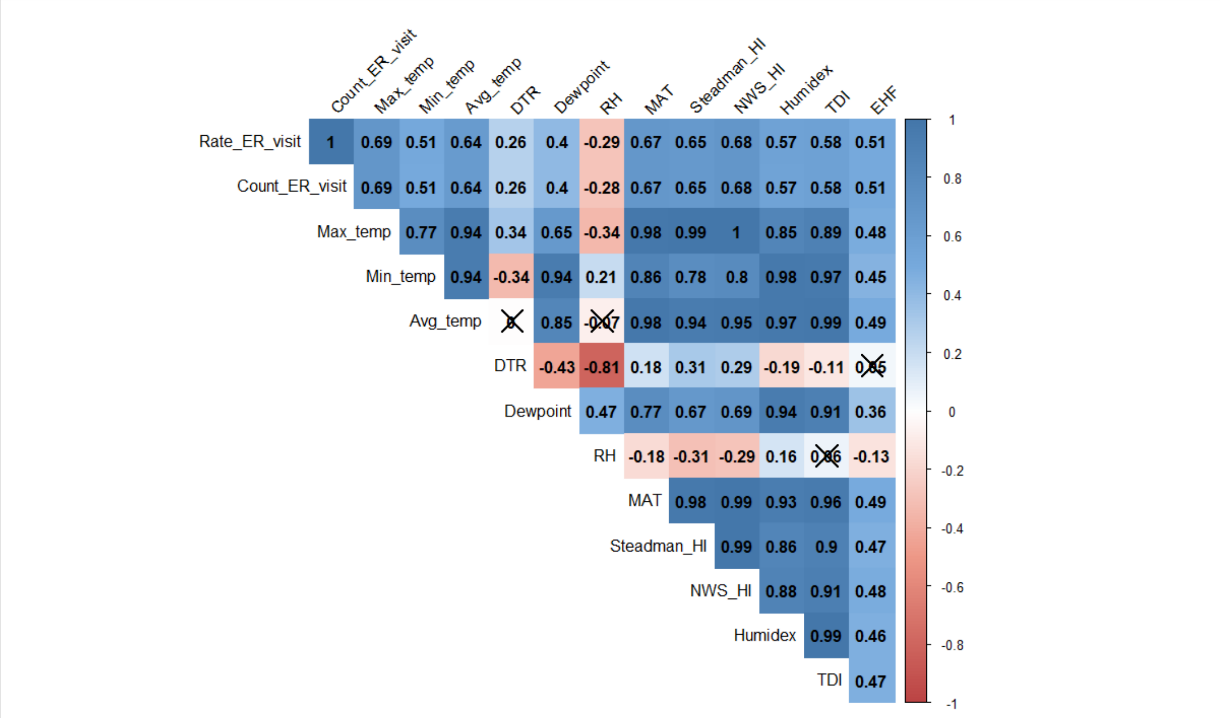

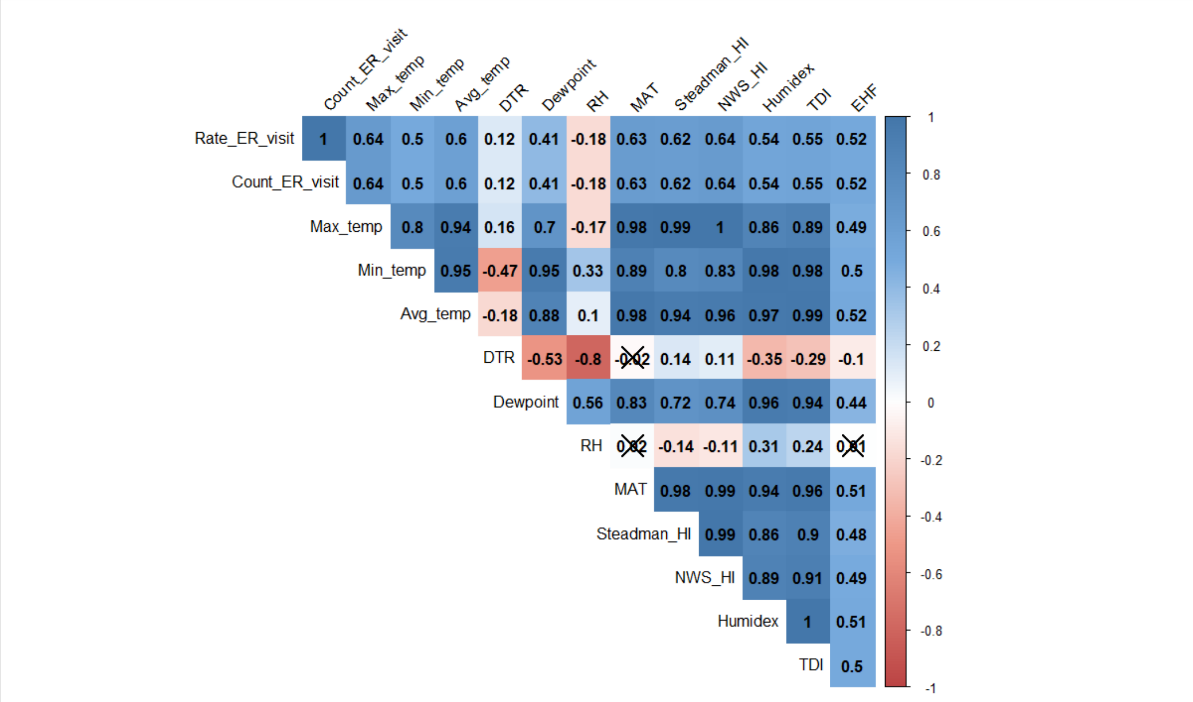


**A**

**B**

**Figure S3-A**. Association between daily maximum temperature and HRI morbidity rate. The nonlinear association was estimated using the GAM framework, using spline terms for temperature and temporal variables (day of week, month, and year) to decompose the temporal trend. The x-axis represents the daily maximum temperature on the centigrade scale, and the y-axis represents the rate of HRI morbidity per 100,000 per day. The solid red and cyan line represent the effect of daily maximum temperature on HRI morbidity for the Coastal and Piedmont regions, respectively. The grey shaded area represents 95% confidence interval. The estimated degree of freedom for Coastal (s=3): 2.86; Piedmont (s=4):3.60.


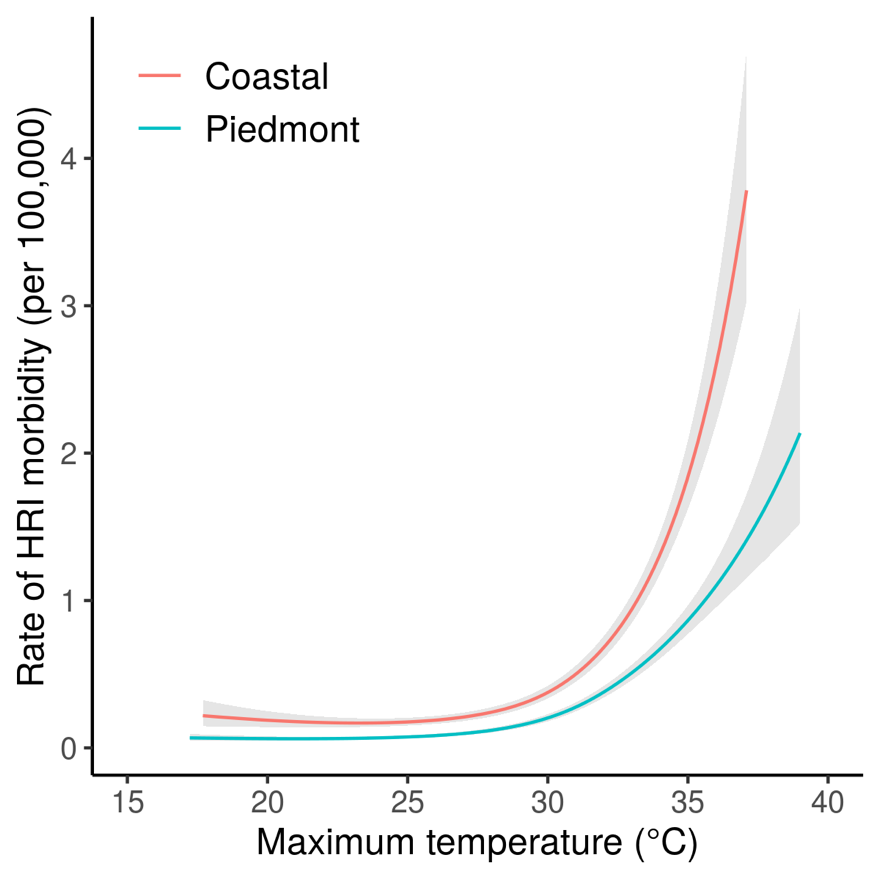


Table S3-B. Generalized additive model - diagnostics. a- based on equation 2; b- based on equation 3.

|  | Coastal |  | Piedmont |  |
| --- | --- | --- | --- | --- |
|  | a | b | a | b |
| AIC | 189 | -783 | -1779 | -1586 |
| Deviance explained (%) | 76.6 | 70.1 | 79.6 | 74.2 |
| R-squared (%) | 77.3 | 54.9 | 76.7 | 69.1 |

**Figure S4.** S 4. Distributive HRI ED visit risk associated with daily maximum temperature (A-Coastal & B-Piedmont). The color gradient represents risk ratio, blue – protective effect, and red – adverse effect. The x-axis represents lag values corresponding to the distributive effect (lag 0: same-day effect and lag 5: risk distributed by 5 days of exposure). The risk ratio values were generated using the DLNM framework.


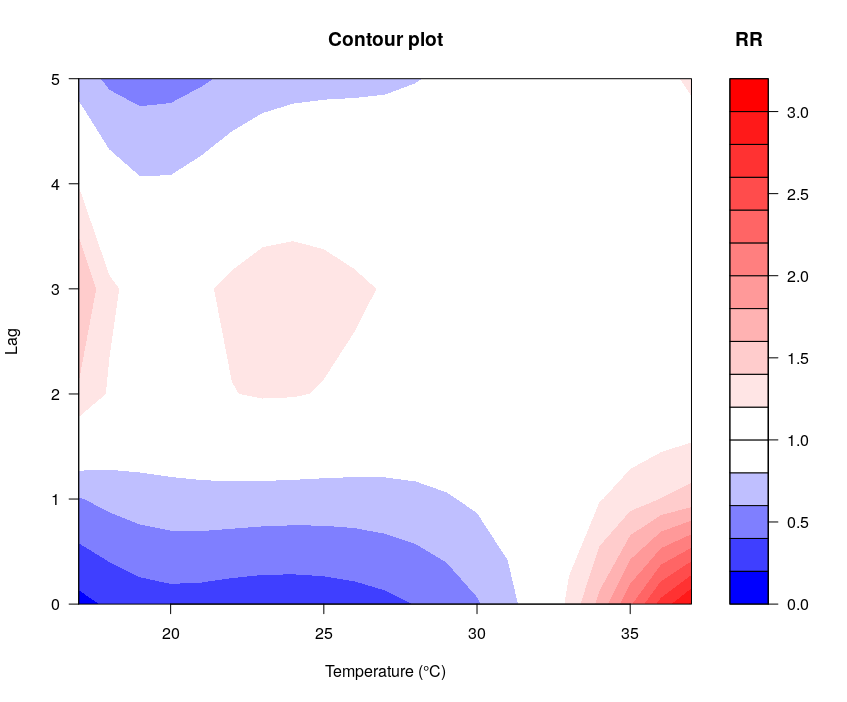

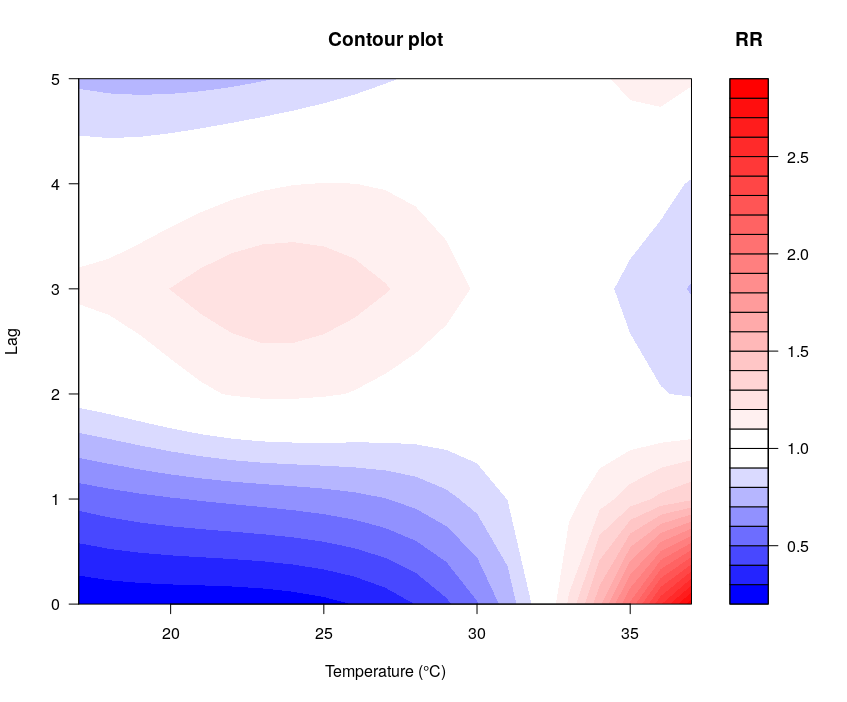


**A**

**B**

**Figure S5.** Distributive HRI morbidity risk associated with daily maximum temperature exposure. This figure was generated using DLNM. Panels A-D are the results for Coastal region and E-H are for Piedmont region. The plots A, B, E, & F shows the association between daily maximum temperature (on x-axis) and HRI morbidity risk (on y-axis). A & E were plotted by holding lag at 0 days (same day exposure-response association). B & F were plotted to show the distributive association between temperature and HRI morbidity at lag 5^th^ day. The panels C, D, G, & H are plotted to show the distributive associations across lag 0-5 days (on x-axis) and HRI morbidity risk (on y-axis). C & G were plotted by holding daily maximum temperature at 32ºC, whereas D & H were plotted by holding daily maximum temperature at 35ºC.


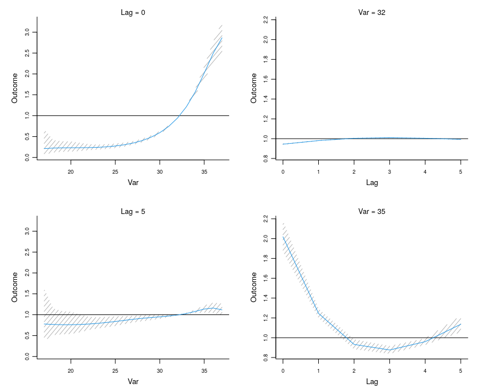

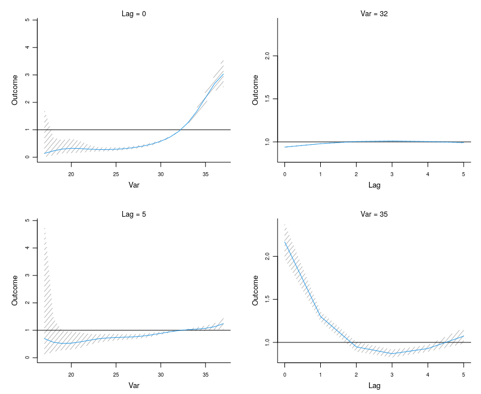


**A**

**H**

**F**

**G**

**E**

**D**

**C**

**B**

**
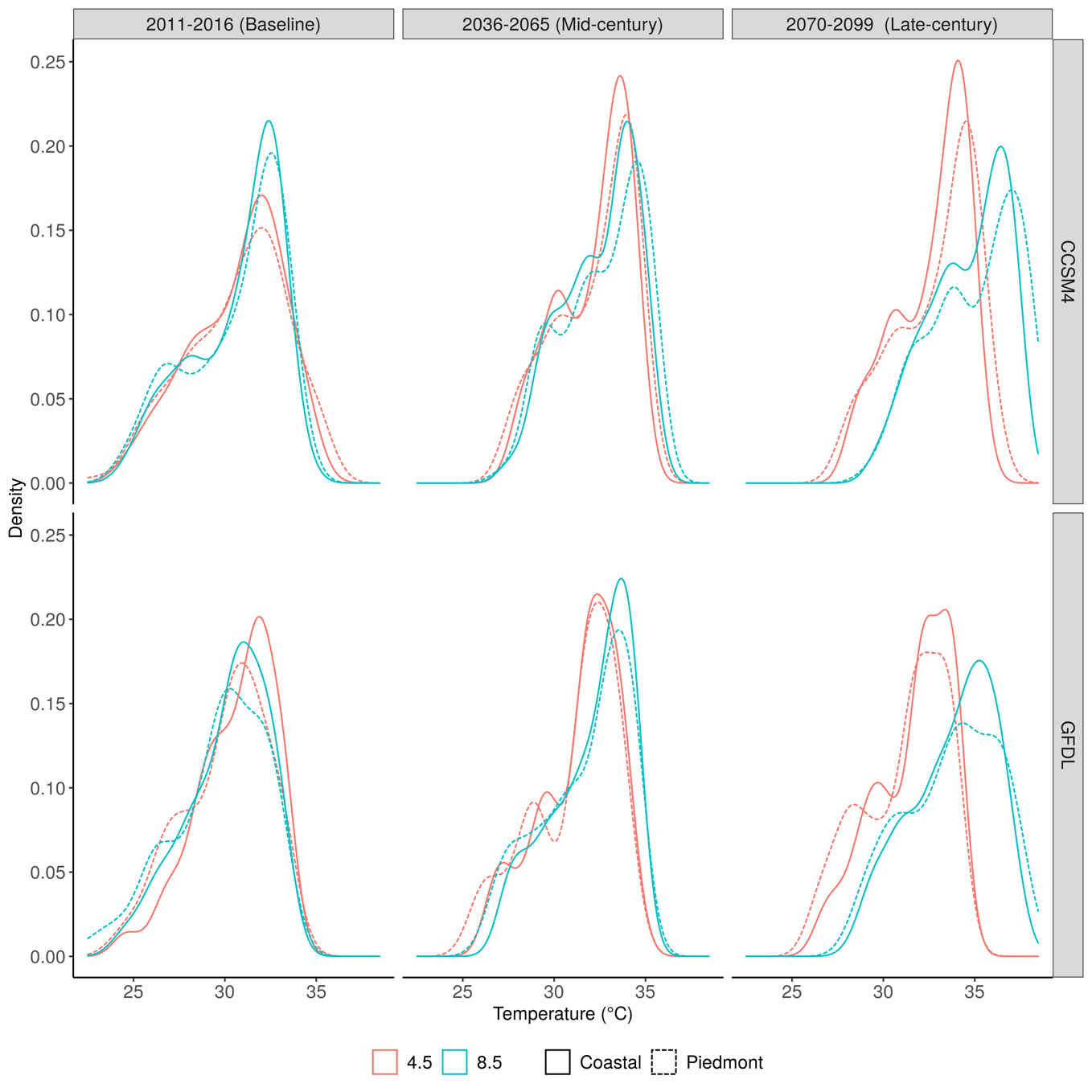
Figure S6.** Projected maximum temperature using CCSM4 and GFDL model simulations. Climate projection data was obtained from the Localized Constructed Analogs database at 1/16º resolution. Among the 32 CIMP5 model runs, we included CCSM4 and GFDL simulations in this study. The x-axis represents temperature on a centigrade scale and y-axis represents probability of distribution.
